# *Lactobacillus acidophilus* inhibits the TNF-α-induced increase in intestinal epithelial tight junction permeability via a TLR-2 and PI3K-dependent inhibition of NF-κB activation

**DOI:** 10.3389/fimmu.2024.1348010

**Published:** 2024-07-16

**Authors:** Mohammad Haque, Lauren Kaminsky, Raz Abdulqadir, Jessica Engers, Evgeny Kovtunov, Manmeet Rawat, Rana Al-Sadi, Thomas Y. Ma

**Affiliations:** ^1^ Department of Medicine, Penn State Milton S. Hershey Medical Center, Hershey, PA, United States; ^2^ Department of Internal Medicine, University of Nebraska Medical Center, Omaha, NE, United States

**Keywords:** *Lactobacillus acidophilus*, TNF-α, NF-κB, TLR-2, MLCK, PI3K, intestinal tight junction barrier

## Abstract

**Background:**

Defective intestinal epithelial tight junction (TJ), characterized by an increase in intestinal TJ permeability, has been shown to play a critical role in the pathogenesis of inflammatory bowel disease (IBD). Tumor necrosis factor-α (TNF-α) is a key pro-inflammatory cytokine involved in the immunopathology of IBD and has been shown to cause an increase in intestinal epithelial TJ permeability. Although TNF-α antibodies and other biologics have been advanced for use in IBD treatment, these therapies are associated with severe side effects and have limited efficacy, and there is an urgent need for therapies with benign profiles and high therapeutic efficacy. Probiotic bacteria have beneficial effects and are generally safe and represent an important class of potential therapeutic agents in IBD. *Lactobacillus acidophilus* (LA) is one of the most used probiotics for wide-ranging health benefits, including in gastrointestinal, metabolic, and inflammatory disorders. A specific strain of LA, LA1, was recently demonstrated to have protective and therapeutic effects on the intestinal epithelial TJ barrier. However, the mechanisms of actions of LA1 remain largely unknown.

**Methods:**

The primary aim of this study was to investigate microbial-epithelial interactions and novel signaling pathways that regulate the effect of LA1 on TNF-α-induced increase in intestinal epithelial TJ permeability, using cell culture and animal model systems.

**Results and Conclusion:**

Pre-treatment of filter-grown Caco-2 monolayers with LA1 prevented the TNF-α-induced increase in intestinal epithelial TJ permeability by inhibiting TNF-α-induced activation of NF-κB p50/p65 and myosin light chain kinase (MLCK) gene and kinase activity in a TLR-2-dependent manner. LA1 produced a TLR-2- and MyD88-dependent activation of NF-κB p50/p65 in immune cells; however, LA1, in intestinal cells, inhibited the NF-κB p50/p65 activation in a TLR-2-dependent but MyD88-independent manner. In addition, LA1 inhibition of NF-κB p50/p65 and MLCK gene was mediated by TLR-2 pathway activation of phosphatidylinositol 3-kinase (PI3K) and IKK-α phosphorylation. Our results demonstrated novel intracellular signaling pathways by which LA1/TLR-2 suppresses the TNF-α pathway activation of NF-κB p50/p65 in intestinal epithelial cells and protects against the TNF-α-induced increase in intestinal epithelial TJ permeability.

## 1 Introduction

Inflammatory bowel disease (IBD) includes Crohn’s disease (CD) and ulcerative colitis (UC) and is characterized by recurrent intestinal inflammation primarily affecting the gastrointestinal tract (1–4). IBD is triggered in part by an inappropriate immune response to gut microbial antigens (5–7). A single cell layer of columnar intestinal epithelial cells connected by tight junctions (TJs) coats the entire intestinal mucosal surface and serves as a protective barrier against the mucosal penetration of luminal contents (2, 8, 9). TJs are located at the apical-most points on the basolateral membrane and form a physical and functional barrier against the paracellular permeation of noxious substances present in the intestinal lumen (including bacteria, bacterial toxins, bacterial by-products, digestive enzymes, and degraded food products) (10–12). The disruption of the intestinal TJ barrier allows paracellular permeation of bacterial antigens and bacterial by-products into the intestinal tissue, leading to the immune activation (1, 13). Patients with IBD have a defective intestinal TJ barrier manifested by an increase in intestinal permeability (14–16). Clinical studies have shown that the phenomenon of “leaky gut” correlates with poor clinical outcomes, whereas therapeutic re-tightening of the intestinal TJ barrier is associated with more rapid clinical improvement and prolonged clinical remission (17–19).

During the active phase of IBD, pro-inflammatory cytokines are released into the gut mucosal compartment, including tumor necrosis factor-α (TNF-α), interleukin (IL)-1β, and interferon (IFN)-γ (20–26). TNF-α plays a central role in the pathogenesis of intestinal inflammation in patients with IBD and, at clinically relevant concentrations, TNF-α causes a marked increase in intestinal permeability (27–34). The inhibition of the TNF-α-induced increase in intestinal permeability has been shown to prevent intestinal inflammation and promote healing in animal models of IBD (23, 35–39). Anti-TNF-α antibody therapy is widely used to treat intestinal inflammation associated with IBD and to maintain clinical remission (40–43). However, these therapies are associated with severe side effects and frequent clinical relapses, and thus, there remains an urgent need for alternative treatment modalities with minimum side effects and high efficacy (44–47). Probiotic bacteria are health-promoting commensal bacteria naturally occurring in the gut with excellent safety profiles and represent an important potential therapeutic option (48–52). The beneficial actions of probiotics may be exerted by up-regulation or maintenance of intestinal epithelial TJ barrier, mucin secretion, modification of gut microbiota composition and activity, protection against oxidative stress and the capability to decrease the risk of accumulation of reaction oxygen metabolites, and reduction of immune response and subsequently reduction of inflammation (53–58). Despite the potential promise, essential limitations related to the use of probiotics exist due to the lack of detailed preclinical and mechanistic studies and precise identification of the specific probiotic strains that have anti-inflammatory biological actions (52). The beneficial effects of gut microbiota are bacterial strain and host-specific (59). Although there is an important recognition that some commensal bacteria have a beneficial role in gut homeostasis by preserving or promoting the intestinal barrier function, it remains unclear as to which probiotic species and strains produce a persistent, predictable enhancement in TJ barrier and which could be advanced to treat intestinal inflammation by targeting the TJ barrier. For example, published studies have indicated that *Lactobacillus acidophilus*, *L. casei*, *L. plantarum*, or *L. rhamnosus* cause a modest enhancement in intestinal epithelial TJ barrier, while others have been shown to have minimal, disruptive, or no effect (60–70). A recent study from our laboratory reported that a particular strain of probiotic bacteria *Lactobacillus acidophilus* (LA), referred to as LA1, caused a unique, marked enhancement of the intestinal epithelial TJ barrier and prevented the development of dextran sulfate sodium (DSS)-induced colitis by preserving the intestinal epithelial TJ barrier (71). In contrast, LA strain LA3 did not affect the intestinal TJ barrier function or protect against the DSS-induced colitis (71). Previous studies have shown that the TNF-α-induced increase in intestinal epithelial TJ permeability is mediated by NF-κB p50/p65 activation of myosin light chain kinase (MLCK) gene and kinase activity in both cell culture and animal model systems (27–30, 32, 33, 72). The major aim of this study was to investigate the strain-specific TJ barrier protective effects of LA1 against the TNF-α-induced increase in intestinal epithelial TJ permeability and to delineate the intracellular mechanisms involved using *in vitro* intestinal epithelial cell and live animal model systems.

Previous studies have shown that the stimulation of the Toll-like receptor-2 (TLR-2) signaling pathway leads to the MyD88-dependent NF-κB p50/p65 activation in immune cells and other cell types in response to bacterial infections (73–76). In the studies described herein, we provide potentially paradigm-shifting finding that in intestinal epithelial cells, and unlike in immune cells, the probiotic bacterial strain LA1 protects the intestinal epithelial TJ barrier by inhibiting NF-κB p50/p65 activation in a TLR-2-dependent and MyD88-independent intracellular signaling process. Our results suggest that the probiotic bacteria in the intestinal mucosal surface may suppress enterocyte NF-κB activation and preserve the intestinal epithelial TJ barrier function by accessing the TLR-2 complex on the enterocyte apical membrane surface.

## 2 Materials and methods

### 2.1 Reagents

Cell culture media [Dulbecco’s Modified Eagle Medium (DMEM)], trypsin, fetal bovine serum (FBS), glutamine, penicillin, streptomycin, and phosphate-buffered saline (PBS) were purchased from GIBCO-BRL (Grand Island, NY). Polyinosinic-polycytidylic acid [poly(I:C)] was purchased from Invivogen (Sand Diego, CA). Peptidoglycan from Bacillus subtilis, Anti-NIK, p-IKK-α, p-IKK-β, p-IκB-α, pMLC, p-NF-κBp65, MLCK, pPI3K were obtained from Sigma-Aldrich (St. Louis, MO). Anti-β-actin-HRP tagged antibody was obtained from Santa Cruz Biotechnology (Dallas, TX). Anti-TLR-2 antibodies were purchased from Abcam (Cambridge, MA). Horseradish peroxidase-conjugated secondary antibodies for Western blot analysis were purchased from Invitrogen (San Francisco, CA). Human and mouse recombinant TNF-α were purchased from R&D systems (Minneapolis, MN).

### 2.2 Determination of Caco-2 epithelial monolayer resistance and paracellular permeability

Caco-2 cells (passage 18) were purchased from the American Type Culture Collection (ATCC) and maintained at 37°C in a culture medium as previously described (27, 71). For filter growth, Caco-2 cells were plated on Transwell plates for 3-4 weeks until the transepithelial resistance (TER) reached 400-500 Ω*cm^2^. Caco-2 paracellular permeability was assessed by measuring the luminal-to-serosal flux rate of a paracellular probe, fluorescein isothiocyanate-labeled FITC dextran 10 kDa (mol wt.: 10,000 g/mol). For determination of mucosal-to-serosal flux rates, a known concentration (25 μg/ml) of FITC dextran 10 kDa was added to the apical solution at the beginning of each experiment. After each experimental period, the solution from the basolateral chamber was collected and measured in a fluorescence microplate reader SpectraMax iD3 (Molecular Devices, San Jose, CA).

### 2.3 Preparation of bacterial culture and cell-free culture supernatant

The *Lactobacillus* strains were purchased from ATCC. With shaking, these bacteria were grown overnight in MRS broth (Difco, Detroit, MI) at 37°C. Live bacteria were spun down by centrifuging at 12,000 × *g* for 10 min, and pellets were stored at −80°C for further experiments. For treating Caco-2 monolayers, the bacterial pellet was suspended in DMEM and diluted to OD600nm = 0.135 (1x10^8^ colony-forming units (CFU)) in the same media and applied to the apical surface of cell monolayers (71).

### 2.4 Determination of mouse small intestinal permeability *in vivo*

The Penn State University Animal Care and Use Committee approved the animal studies. Wild-type (WT) mice (C57BL/6 background), TLR-2 deficient mice (TLR-2^-/-^), and MyD88 deficient mice (MyD88^-/-^) were obtained from The Jackson Laboratory (Bar Harbor, Maine). Mice were treated with 5 μg of TNF-α by intraperitoneal injection (i.p.) (27, 28) and the LA effect on intestinal permeability was determined *in vivo* using a re-cycling intestinal perfusion method (77, 78). For *in vivo* studies, 1 × 10^9^ CFU of LA in 200 μl PBS was administered 24 hr before TNF-α treatment by oral-gastric gavage, and mouse intestinal permeability was measured after 24 hr of TNF-α treatment. Mice were anesthetized, and a 6-10 cm segment of mouse small intestine was isolated and cannulated with a small diameter plastic tube and continuously perfused with 5 ml Krebs-phosphate saline buffer for a 2 hr perfusion period. An external recirculating pump was used to recirculate the perfusate at a constant flow rate (0.75 ml/min). The intestinal permeability was assessed by measuring the luminal-to-serosal flux rate of the paracellular probe Texas Red-labeled dextran 10kDa (MW = 10,000 g/mol).

### 2.5 Western blot analysis

Caco-2 monolayers were treated with 10 ng/ml TNF-α for various time periods. At the end of the experimental period, Caco-2 monolayers were immediately rinsed with ice-cold PBS, and cells were lysed with RIPA lysis buffer (Sigma-Aldrich, St. Louis, MO). Laemmli gel loading buffer was added to the lysate that contained 20 to 50 µg of protein and boiled for 7 minutes, after which time proteins were separated on SDS-PAGE gel. Proteins from the gel were transferred to a membrane (Trans-Blot Transfer Medium, Nitrocellulose Membrane; Bio-Rad Laboratories) overnight. The membrane was incubated for 2 hours in a blocking solution (5% dry milk in tris-buffered saline/Tween 20 buffer). The membrane was incubated with proper primary antibodies in a blocking solution. After being washed in tris-buffered saline/1% Tween buffer, the membrane was incubated in appropriate secondary antibodies and developed with the Biorad ChemiDoc Imaging System (BioRad, Hercules, California).

### 2.6 Immunofluorescence study

Intestinal tissues were collected from the mice at the indicated time points. Samples were collected in embedding cassettes and blocked with 10% neutral buffered formalin. Samples were infiltrated with wax, and infiltrated tissues were embedded into wax blocks. For immunostaining, sections were de-parafinized with 3 washes of xylene. Caco-2 monolayers were fixed on filters with ice-cold methanol. Antigen unmasking was done according to manufacturer protocol, and samples were incubated for 2 hr with anti-NF-κB p65 antibody (Santa Cruz) or anti-pMLC antibody (Cell Signaling Technology, Danvers, MA), Alexa conjugated, or Cy3 secondary antibody (Cell Signaling Technology; Danvers, MA) was used to detect the primary antibodies. All the primary and secondary antibodies were used at the concentrations suggested by the manufacturers. ProLong Gold antifade reagent (Invitrogen), containing DAPI as a nuclear stain, was used to mount the samples on glass slides. Samples were imaged using the C2+ confocal microscope system (Nikon) and ZEISS Axio Imager.M2 Microscope with images processed with ZEN 3.2 (Blue edition) software.

### 2.7 Immunostaining of splenocytes

Spleens were harvested from wild-type (WT), TLR-2^-/-^, and MyD88^-/-^ mice. Splenocytes were processed into single-cell suspensions and plated on coverslips in 6-well plates. Following adherence to coverslips, splenocytes were treated with LA or TNF-α for 30 minutes, then fixed in methanol for 20 minutes at -20°C. Cells were permeabilized with 0.1% Triton X-100 in PBS for 10 minutes and blocked in 10% NDS for 1 hr. Staining with anti-NF-κB p65 antibody (1:300; Santa Cruz Biotechnology; Dallas, TX) in 1% NDS for 1 hour was followed by incubation with secondary antibody, goat anti-mouse IgG-FITC (1:1000; Invitrogen; Waltham, MA) in 1% NDS for 1 hr. ProLong Gold Antifade reagent was used to mount coverslips on slides. Sections were imaged using the C2+ confocal microscope system (Nikon) or with Nikon Fluorescent microscope with images minimally processed using NIS Elements software and were processed with ZEN 3.2 (Blue edition) software.

### 2.8 RNA isolation and reverse transcription

Caco-2 cells (5 × 10^5^/filter) were seeded into 6-well transwell permeable inserts and grown to confluence. Filter-grown Caco-2 cells were treated with proper experimental reagents for desired periods. At the end of the experimental period, cells were washed twice with ice-cold PBS. Total RNA was isolated using Qiagen RNeasy Kit (Qiagen, Valencia, CA, USA) according to the manufacturer’s protocol. Total RNA concentration was calculated at 260 nm by NanoDrop One Spectrophotometer (Thermofisher Scientific, Waltham, MA). The reverse transcription (RT) was carried out using the GeneAmp Gold RNA PCR core kit (Applied Biosystems, Foster City, CA). Two micrograms of total RNA from each sample were reverse transcribed into cDNA in a 40-μl reaction containing 1× RT-PCR buffer, 2.5 mM MgCl2, 250 μM of each dNTP, 20 U RNase inhibitor, 10 mM DL-Dithiothreitol (DTT), 1.25 μM random hexamer and 30 U multiscribe RT. The RT reactions were performed in a thermocycler (PTC-100, MJ Research, Waltham, MA, USA) at 25°C for 10 min., 42°C for 30 min., and 95°C for 5 min.

### 2.9 Quantification of gene expression using real-time PCR

The real-time PCRs were carried out using Taqman universal PCR master mix kit (Applied Biosystems, Branchburg, NJ, USA) as previously described (9). Each real-time PCR reaction contained 10 μl RT reaction mix, 25 μl 2× Taqman universal PCR master mix, 0.2 μM probe and 0.6 μM primers. Primer and probe design for the real-time PCR was made with Primer Express version 2 from Applied Biosystems. [The MLCK specific primer pairs consisted of 5′-AGGAAGGCAGCATTGAGGTTT-3′[forward], 5′-GCTTTCAGCAGGCAGAGGTAA-3′[reverse]; probe specific for MLCK consisted of FAM 5′-TGAAGATGCTGGCTCC-3′ TAMRA; the internal control glyceraldehyde 3-phosphate dehydrogenase (GAPDH)-specific primer pairs consisted of 5′-CCACCCATGGCAAATTCC-3′[forward], 5′-TGGGATTTCCATTGATGACCAG-3′[reverse]; probe specific for GAPDH consisted of JOE 5′-TGGCACCGTCAAGGCTGAGAACG-3′ TAMRA].

### 2.10 Cloning of the full-length MLCK promoter region and luciferase assay

The full-length (FL) MLCK promoter region (2091-bp) (accession# DQ090939) was cloned using Genome Walker system (Clontech, Mountain View, CA, USA) (9). FL MLCK promoter reporter plasmids were transfected using the pGL-3 basic luciferase reporter vector. The primers used for cloning the FL MLCK promoter construct are 5’GCCGGTACCGAGAAGCAGGAGAGTATTAAATG3’. DNA construct of MLCK promoters was transiently transfected into Caco-2 cells using transfection reagent lipofectamine 2000 (Life Technologies). Renilla luciferase vector (pRL-TK, Promega) was cotransfected with the plasmid construct as an internal control, as previously described (9, 33). After specific treatment, Caco-2 cells were washed twice with 1 ml ice-cold PBS, followed by the addition of 400 μl 1× passive lysis buffer, incubated at room temperature for 15 min, scraped and transferred into an Eppendorf tube, and centrifuged for 15 s at 13,000 rpm in a microcentrifuge. Luciferase activity was determined using the dual luciferase assay kit (Promega). Twenty microliters of the supernatant were used for each assay. Luciferase values were determined, and the value of reporter luciferase activities was then divided by that of Renilla luciferase activities to normalize for differences in transfection efficiencies. The average activity value of the control samples was set to 1.0. The luciferase activity of the MLCK promoter in treated samples was determined relative to the control samples.

### 2.11 Statistical analysis

The statistical significance of differences between mean values was assessed using students’ t-tests for unpaired data and analysis of variance analysis whenever needed. All reported significance levels represent two-tailed P values. P < 0.05 was used to show statistical significance. All *in vitro* experiments that used Caco-2 monolayers, including assessment of the TJ barrier function and biochemical and molecular studies, were performed in triplicates or quadruplicates and were repeated at least three times for reproducibility. The immunoblot analysis and cell imaging studies were repeated three to four times. The densitometry analysis was performed using ImageJ 1.54g. The animal studies were performed individually, and each experimental group consisted of three to six animals.

## 3 Results

### 3.1 *Lactobacillus acidophilus* strain LA1 inhibits the TNF-α-induced increase in Caco-2 TJ permeability

**Figure 1:**
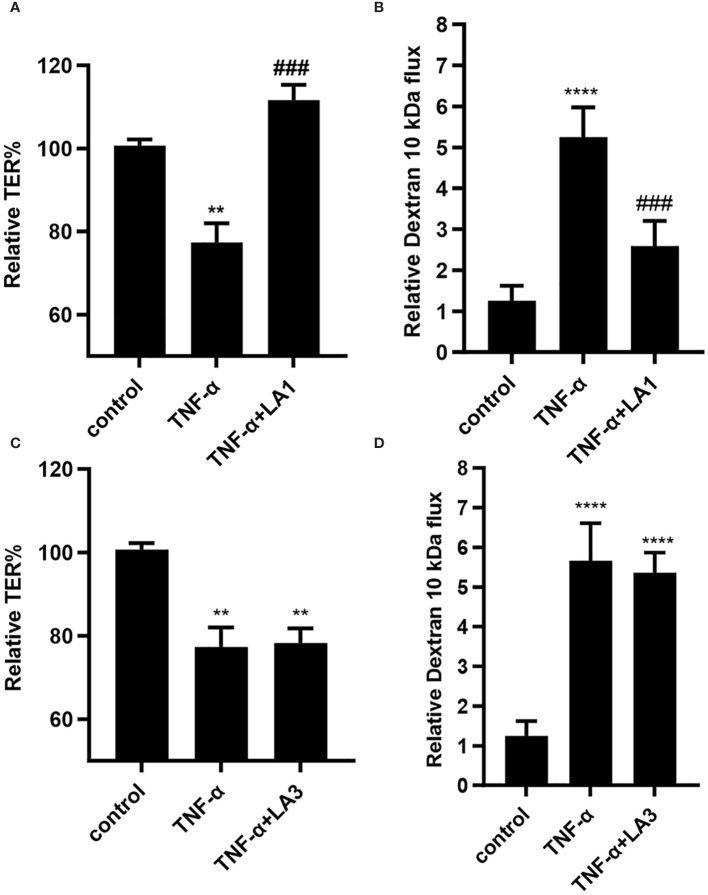
Effect of *Lactobacillus acidophilus* strains, LA1 and LA3, on TNF-α-induced increase in filter-grown Caco-2 TJ permeability. **(A)** Strain LA1 prevented the TNF-α-induced drop in Caco-2 TER. LA1 (1x10^8^ CFU/ml for 24 hr); TNF-α (10 ng/ml for 24 hr); LA1 pre-treated 2 hr prior to TNF-α treatment. **, *P*<0.01 vs. control; ^###^, *P*<0.001 vs. TNF-α treatment. **(B)** LA1 prevented the TNF-α-induced increase in mucosal-to-serosal flux of paracellular marker dextran 10 kDa; ****, *P*<0.0001 vs. control; ^###^, *P*<0.001 vs. TNF-α treatment. **(C)** Strain LA3 did not affect the TNF-α-induced drop in Caco-2 TER or **(D)** the TNF-α-induced increase in Caco-2 TJ permeability to dextran 10 kDa. **, *P*<0.01 vs. control ****, *P*<0.0001 vs. control.

The effect of *Lactobacillus acidophilus* (LA1 or LA3) on TNF-α-induced increase in intestinal epithelial TJ permeability was examined in filter-grown Caco-2 monolayers. TNF-α (10 ng/ml) addition to the basolateral compartment produced a drop in Caco-2 TER (~25% drop) and an increase (~5-fold increase) in trans-epithelial flux of paracellular marker, FITC-labeled dextran 10 kDa, in filter-grown Caco-2 monolayers over the 24-hr experimental period (**Figures 1A, B**). As previously reported (71), LA1 (1x10^8^ CFU/ml) addition to the apical membrane resulted in an enhancement in Caco-2 TJ barrier function with an 80% increase in Caco-2 TER and a corresponding decrease in luminal-to-serosal dextran flux (45% decrease) while LA3 had no effect (data not shown). Based on previous studies that found the beneficial concentrations of probiotic bacterial species to range from 1 × 10^7^ to 1 × 10^9^ CFU/ml, bacterial concentration of 1 × 10^8^ CFU/ml were used in Caco-2 cells in the current studies (71, 79, 80). We also performed a dose-dependent effect of LA in Caco-2 monolayers to verify the most beneficial dose of LA on the intestinal TJ barrier. We found that a 1x108 CFU/ml bacterial concentration exerted the maximal effect on Caco-2 TJ barrier (data not shown). The LA1 addition inhibited the TNF-α-induced decrease in Caco-2 TER and increase in dextran flux (**Figures 1A, B**). In contrast, LA3 did not inhibit the TNF-α effect on Caco-2 TER or dextran flux (**Figures 1C, D**), indicating that LA1 causes a strain-specific inhibition of TNF-α-induced increase in Caco-2 TJ permeability.

### 3.2 LA1 prevents the TNF-α-induced increase in Caco-2 TJ permeability by suppressing NF-κB p50/p65 activation

**Figure 2:**
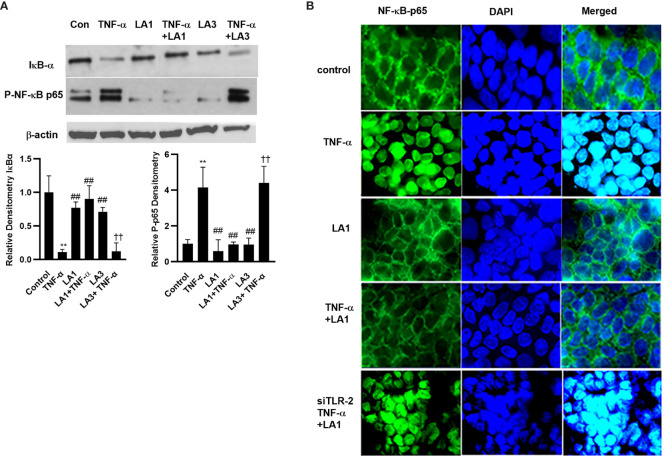
Effect of LA1 on TNF-α activation of NF-κB p65 in Caco-2 monolayers. **(A)** LA1 treatment (1x10^8^ CFU/ml) did not affect the activation of NF-κB p65 as assessed by degradation of IκB-α and phospho-p65 expression in Caco-2 monolayers (30-min experimental period). TNF-α (10 ng/ml) caused a rapid increase in phospho-p65 expression and degradation of IκB-α in Caco-2 monolayers (30-min experimental period). LA1, but not LA3, prevented the TNF-α-induced activation of NF-κB p65. Relative densitometry of IκB-α and p-p65 indicated below; **, *P*<0.01 vs. control; ^##^, *P*<0.01 vs. TNF-α treatment; ^††^, *P*<0.01 vs. LA3 treatment. **(B)** LA1 did not affect the cytoplasmic-to-nuclear translocation of NF-κB p65 in Caco-2 monolayers but inhibited the TNF-α-induced nuclear translocation of NF-κB p65 (30-min experimental period). TLR-2 siRNA transfection (48 hr) prevented the LA1 inhibition of TNF-α-induced nuclear translocation of NF-κB p65 as determined by immunofluorescence imaging. NF-κBp65: green; Nuclei: blue; 60x (Nikon fluorescent microscopy).

The TNF-α-induced increase in intestinal epithelial TJ permeability is mediated by a canonical pathway activation of nuclear transcription factor NF-κB p50/p65 (27, 28, 33, 81, 82). The activation of NF-κB p50/p65 requires inhibitory-κB kinase (IKK) induced phosphorylation and proteasomal degradation of IκB-α, leading to NF-κB p50/p65 activation and nuclear translocation (83–86). In the following studies, the effect of LA1 on TNF-α-induced activation of NF-κB p50/p65 was examined. The TNF-α treatment caused degradation of IκB-α, phosphorylation of NF-κB p65 subunit (Ser536) (**Figure 2A**), and subsequent nuclear translocation of NF-κB p65 in Caco-2 monolayers (**Figure 2B**). As shown in the immunostaining studies, TNF-α treatment caused NF-κB p65 translocation to the nucleus (**Figure 2B**). LA1 treatment by itself did not activate the basal level of IκB-α or NF-κB activation (**Figure 2B**). However, pre-treatment with LA1 almost completely inhibited the TNF-α-induced degradation of IκB-α and NF-κB activation (**Figure 2**). LA3 did not prevent the TNF-α-induced activation of NF-κB p65 as measured by the phosphorylation of NF-κB p65 and degradation of IκB-α, (**Figure 2A**).

### 3.3 LA1 stimulates NF-κB p50/p65 activation in mouse immune cells in a TLR-2-dependent manner

**Figure 3:**
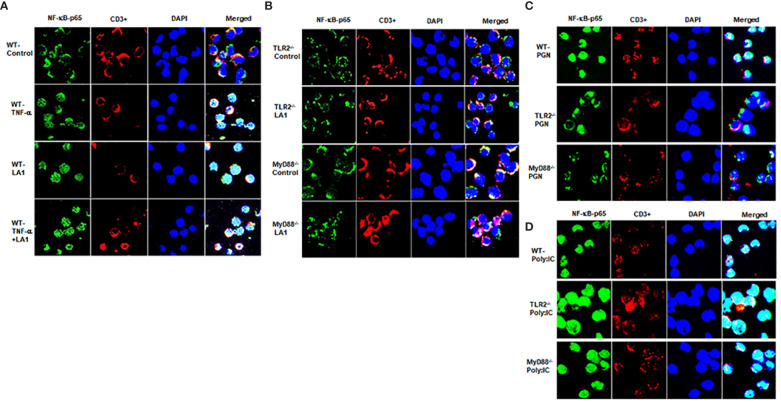
Effect of LA1 on NF-κB activation in mouse splenocytes isolated from wild-type (WT), TLR-2^-/-^ and MyD88^-/-^ mice. Immunofluorescence images showing: **(A)** TNF-α (10 ng/ml) and LA1 (1x108 CFU/ml) treatments caused cytoplasmic-to-nuclear translocation of NF-κB p65 (30-min experimental period) in splenocytes isolated from WT mice (WT, wild-type); **(B)** LA1 did not cause nuclear translocation of NF-κB p65 in immune cells from TLR-2^-/-^ or MyD88^-/-^ mice (30-min experimental period).; **(C)** Peptidoglycan (PGN) (30 µg/ml) caused nuclear translocation of NF-kB p65 in immune cells from WT, but not in immune cells from TLR-2^-/-^ or MyD88^-/-^ mice (30-min experimental period); **(D)** Poly: IC (100 μg/ml) caused nuclear translocation of NF-kB p65 in immune cells from WT, TLR-2^-/-^ and MyD88^-/-^ mice (30-min experimental period).NF-κBp65: green; T-cell marker CD3: red; Nuclei: blue; 60x (Nikon fluorescent microscopy).

Expanding on the above studies showing LA1 inhibition of TNF-α-induced activation of NF-κB p50/p65 in intestinal epithelial cells, in the next studies, we examined the effect of LA1 on NF-κB activation in immune cells. Toll-like receptors (TLRs) have a crucial role in immune response against bacterial invasion (87, 88). In response to pathogen invasion, plasma membrane-associated TLRs, including TLR-2, TLR-4, and TLR-5, recognize pathogen-associated molecular patterns (PAMPs) and cause NF-κB p50/p65 activation via a MyD88-dependent process (74–76). In the following studies, mouse immune cells were obtained from freshly resected mouse spleen. The splenocytes (consisting of lymphocytes, monocytes, and macrophages) were isolated from the mouse spleen as described in the methods section. The LA1 effect on NF-κB p50/p65 activation in mouse splenocytes was assessed by NF-κB p65 nuclear translocation. The CD3^+^ T-cells were identified by antibody labeling and immunostaining. In the untreated control immune cells, NF-κB p65 was localized in the cytoplasm (**Figure 3A**). The LA1 addition caused a rapid (within 30 minutes) cytoplasmic-to-nuclear translocation of NF-κB p65 in both CD3^−^ immune cells and CD3^+^ T-cells (**Figure 3A**), indicating NF-κB activation. The TNF-α treatment of splenocytes also caused nuclear translocation of NF-κB p65 in both CD3^−^ immune cells and CD3^+^ T cells (**Figure 3A**). Next, the regulatory requirement of TLR-2 or MyD88 in LA1 activation of NF-κB was determined in TLR-2 or MyD88 deficient immune cells (obtained from global TLR-2 or MyD-88 knock-out mice, respectively). LA1 did not induce NF-κB activation in immune cells obtained from TLR-2 ^-/-^ or MyD88 ^-/-^ mice (**Figure 3B**), confirming that the LA1 induced NF-κB p50/p65 nuclear translocation in immune cells was dependent on TLR-2 and MyD88. Together, these data indicated that LA1 activates NF-κB p50/p65 in immune cells in a TLR-2- and MyD88-dependent manner. On the other hand, TNF-α is known to activate NF-κB by binding to its receptors TNFRI and II, but not to TLRs (89, 90). The signaling pathway triggered by TNF-α is known to activate NF-κB in a TRAF/TRADD-dependent pathway and MyD88-independent pathway. TNF-α treatment caused NF-κB activation in immune cells obtained from TLR-2 ^-/-^ and MyD88 ^-/-^ mice (data not shown), indicating that TNF-α activation of NF-κB in immune cells is independent on TLR-2 and/or MyD88. We also showed that peptidoglycan (PGN), an activator of NF-κB in a TLR-2 and MyD88-dependent manner (91, 92), caused nuclear translocation of NF-κB p65 in immune cells obtained from WT mice, but not in in immune cells obtained from TLR-2 ^-/-^ and MyD88 ^-/-^ mice (**Figure 3C**). In contrast, polyinosinic-polycytidylic acid [poly(I:C)], a PAMP known to activate NF-κB in a TLR-2 and MyD88-independent manner (93, 94), caused NF-κB p65 nuclear translocation in immune cells obtained from WT, TLR-2^-/-^ and MyD88^-/-^ mice (**Figure 3D**).

### 3.4 LA1 inhibits the NF-κB p50/p65 activation in intestinal epithelial cells in a TLR-2-dependent manner

**Figure 4:**
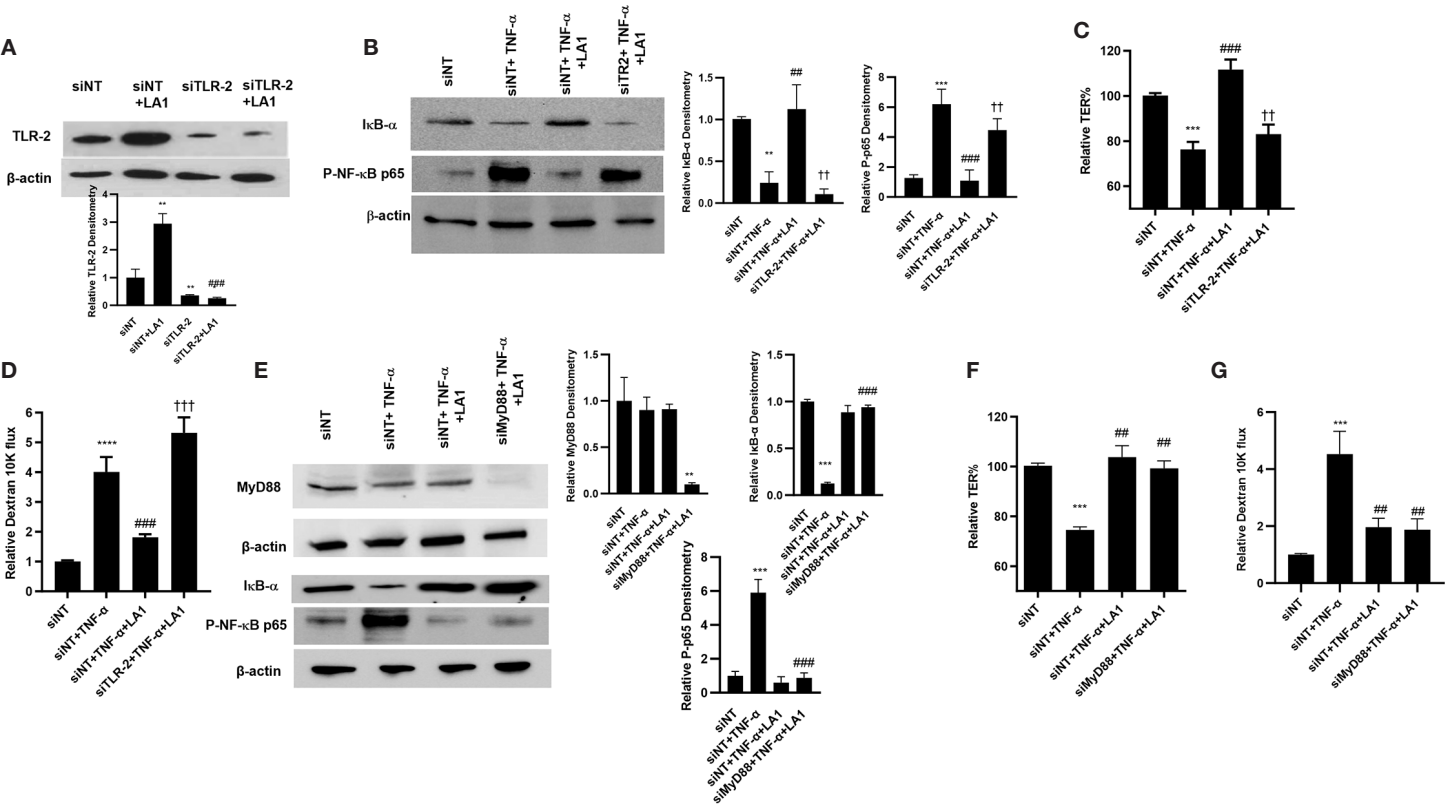
Intracellular mechanisms involved in LA1 protection against TNF-α-induced increase in Caco-2 intestinal epithelial TJ permeability. **(A)** TLR-2 siRNA transfection (48 hr) prevented the LA1-induced increase in TLR-2 protein expression (24 hr treatment). (siNT: siRNA non-target transfection). SiRNA transfection was performed for 48 hr prior to LA1 or TNF-α treatments. Relative densitometry of TLR-2 indicated below; **, *P*<0.01 vs. siNT; ^###^, *P*<0.001 vs. LA1 treatment. **(B)** TLR-2 siRNA transfection prevented the LA1 inhibition of TNF-α-induced degradation of IκB-α and phosphorylation of NF-κB p65 (30-min experimental period). Relative densitometry of IκB-α and p-p65 indicated right; **, *P*<0.01 vs. siNT; ^##^, *P*<0.01 vs. TNF-α treatment; ^††^, *P*<0.01 vs. TNF-α+LA1 treatment. **(C)** TLR-2 siRNA transfection prevented the LA1 inhibition of TNF-α-induced decrease in Caco-2 TER (24hr experimental period), ***, *P*<0.001 vs. control; ^###^, *P*<0.001 vs. TNF-α treatment; ^††^, *P*<0.01 vs TNF-α with LA1 pre-treatment) and **(D)** increase to dextran 10 kDa flux (24hr experimental period); **, *P*<0.01 vs. control; ^###^, *P*<0.001 vs. LA1 treatment; **** *P*<0.0001 vs control; ^†††^, *P*<0.001 vs. TNF-α with LA1 pre-treatment. **(E)** Knocking down MyD88 by siRNA transfection (48 hr) caused a significant depletion of MyD88 expression, but did not prevent the LA1 inhibition of TNF-α-induced degradation of IκB-α and phosphorylation of NF-κB p65 (30-min experimental period). Relative densitometry of MyD88, IκB-α and p-p65 indicated right; **, *P*<0.01 vs. siNT, TNF-α, and TNF-α+LA1 treatments; ***, *P*<0.001 vs. siNT; ^###^, *P*<0.001 vs. TNF-α treatment. **(F)** SiRNA MyD88 transfection did not prevent the LA1 inhibition of TNF-α-induced decrease in Caco-2 TER or **(G)** increase to dextran 10 kDa flux (24 hr experimental period); ***, *P*<0.001 vs. control; ^##^, *P*<0.01 vs. TNF-α treatment.

In the following studies, the knock-down of TLR-2 by siRNA on LA1 inhibition of NF-κB p50/p65 activation in Caco-2 monolayers was determined. As shown in **Figure 2**, LA1 inhibited the TNF-α-activation of NF-κB in Caco-2 intestinal epithelial cells. The silencing of TLR-2 by siRNA (**Figure 4A**) prevented the LA1 inhibition of TNF-α-induced IκB-α degradation, NF-κB p65 phosphorylation (**Figure 4B**) and NF-κB p65 nuclear translocation (**Figure 2B**) as well as the increase in Caco-2 TJ permeability (**Figures 4C, D**). Interestingly, knocking-down MyD88 (**Figure 4E**) did not alter the LA1 inhibition of NF-κB p50/p65 activation (**Figure 4E**) or the increase in Caco-2 intestinal epithelial TJ permeability (**Figures 4F, G**), suggesting that the TLR-2 pathway inhibition of NF-κB activation in Caco-2 cells was MyD88-independent. In combination, these findings suggested that LA1 activates NF-κB p50/p65 in immune cells in a TLR-2 and MyD88-dependent process, whereas LA1 inhibits the TNF-α induced activation of NF-κB p50/p65 in intestinal epithelial cells in a TLR-2-dependent-but MyD88-independent process.

### 3.5 PI3 kinase mediates the TLR-2 pathway inhibition of NF-κB activation

**Figure 5:**
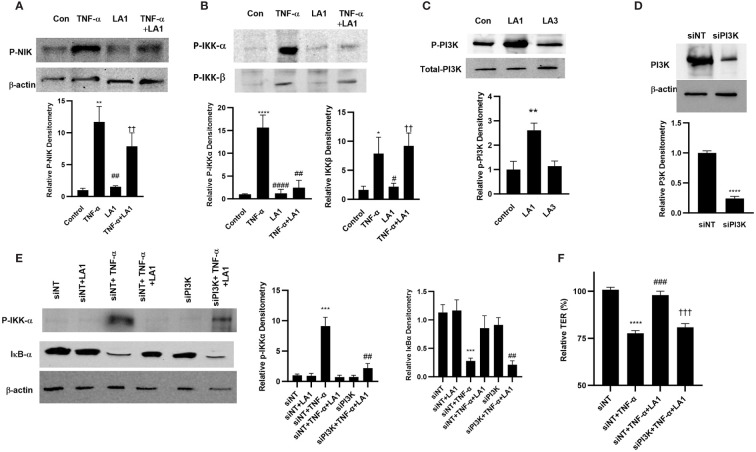
Effect of LA1 inhibition on TNF-α activation of upstream signaling kinases in Caco-2 monolayers. **(A)** LA1 did not prevent the TNF-α-induced activation of NIK as assessed by phospho-NIK (30-min experimental period). Relative densitometry of P-NIK indicated below; **, *P*<0.01 vs. control; ^##^, *P*<0.01 vs. TNF-α treatment; ^††^, *P*<0.01 vs. LA1 treatment. **(B)** LA1 prevented the TNF-α-induced activation of IKK-α, but not IKK-β, as assessed by phospho-IKK-α/IKK-β (30-min experimental period). Relative densitometry of p-IKK-α and p-IKK-β indicated below; *, *P*<0.05 vs. control ****, *P*<0.0001 vs. control; ^#^, *P*<0.05; ^##^, *P*<0.01; ^####^, *P*<0.0001 TNF-α treatment;^††^, *P*<0.01 vs. LA1 treatment. **(C)** LA1, but not LA3, caused activation of PI3K as assessed by phospho-PI3K (30-min experimental period). Relative densitometry of p-PI3K indicated below; **, *P*<0.01 vs. control. **(D)** SiRNA-induced knock-down of PI3K resulted in a significant depletion of PI3K expression (48 hr). Relative densitometry of PI3K indicated below; ****, *P*<0.0001 vs. siNT. **(E)** Knocking down PI3K by siRNA transfection prevented the LA1 inhibition of TNF-α-induced phosphorylation of IKK-α or degradation of IκB-α. SiRNA transfection was performed for 48 hr prior to LA1 treatment for 30 min. Relative densitometry of p-IKK-α and IκB-α indicated right; ***, *P*<0.001 vs. siNT; ^##^, *P*<0.01 vs.TNF-α +LA1 treatment. **(F)** Knocking PI3K by siRNA prevented the LA1 inhibition of TNF-α induced drop in Caco-2 TER. SiRNA transfection was performed for 48 hr prior to LA1 treatment for 24 hr; ****, *P*<0.0001 vs. control; ^###^, *P*<0.001 vs. LA1 or TNF-α treatment; ^†††^, *P*<0.001 vs TNF-α with LA1 pre-treatment.

Next, we investigated the crosstalk between TLR-2 and TNF-α signal transduction pathways and the protein kinase that mediates the LA1 inhibition of TNF-α effect on NF-κB activation and on intestinal TJ permeability. The TNF-α activation of NF-κB p50/p65 is regulated up-stream by mitogen-activated protein-3 kinase (MAP3K) NF-κB-inducing kinase (NIK) phosphorylation and activation of IκB kinases, IKK-α and IKK-β (28). TNF-α caused activation and phosphorylation of NIK (Thr-559) in Caco-2 cells as assessed by phospho-NIK immunoblotting (**Figure 5A**); LA1 did not prevent the TNF-α activation of NIK, suggesting that LA1 does not inhibit NF-κB p50/p65 activation at the level of NIK. TNF-α also caused activation of both IKK-α and IKK-β catalytic subunits (**Figure 5B**); LA1 prevented the TNF-α-induced activation and phosphorylation of IKK-α (Ser-176), but not IKK-β phosphorylation (Ser-180) (**Figure 5B**), demonstrating that LA1 inhibits the IKK-α phosphorylation without affecting IKK-β phosphorylation. Phosphatidylinositol 3-kinase (PI3K) is known to be activated by the TLR-2 signal transduction pathway (95–101). Next, the possibility that PI3K acts as an upstream protein kinase that mediates the LA1/TLR-2 inhibition of TNF-α-induced IKK-α and NF-κB activation was examined. LA1caused PI3K (Ser-361) phosphorylation in Caco-2 monolayers, while LA3 did not (**Figure 5C**). Silencing PI3K by siRNA (**Figure 5D**) prevented the LA1 inhibition of TNF-α-induced IKK-α phosphorylation and IκB-α degradation (**Figure 5E**) and decrease in Caco-2 TER (**Figure 5F**).

### 3.6 LA1 inhibits the MLCK gene activity and kinase activity induced by TNF-α

**Figure 6:**
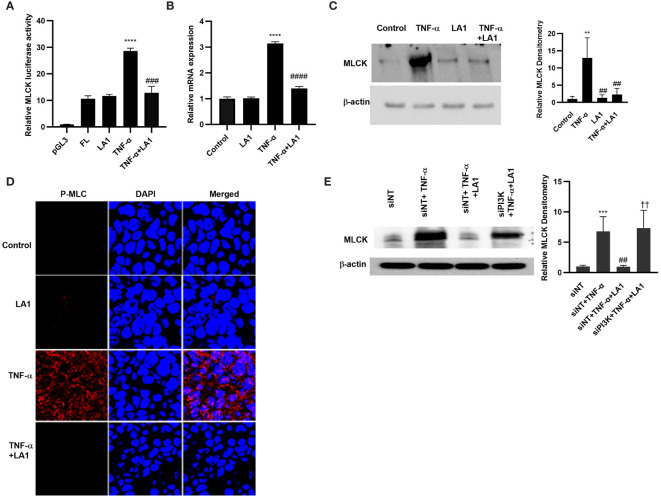
Effect of LA1 on TNF-α activation of MLCK gene, protein expression and activity in Caco-2 monolayers. **(A)** LA1 (1x10^8^ CFU/ml) prevented the TNF-α-(10 ng/ml) induced increase in MLCK gene. pGL-3 basic vector containing the MLCK promoter region was transfected into the filter-grown Caco-2 cells. The MLCK promoter activity was determined by the luciferase assay and expressed as relative luciferase activity; ****P < 0.0001 vs. Full-Length (FL); ^###^, *P*<0.001 vs. TNF-α treatment. **(B)** LA1 inhibited the MLCK TNF-α induced increase in MLCK mRNA levels; ****, *P* < 0.0001 vs. control; ^####^, *P*<0.0001 vs. TNF-α treatment, **(C)** protein expression (Relative densitometry of MLCK indicated right; ^**^, *P*<0.01 vs. control; ^##^, *P*<0.01 vs.TNF-α) and **(D)** MLCK kinase activity as assessed by phospho-MLC (P-MLC) immunofluorescence imaging (24 hr experimental period). P-MLC: red; Nuclei: blue; 63x (Confocal microscopy). **(E)** Knocking down PI3K by siRNA transfection (48 hr) prevented the LA1 inhibition of TNF-α-induced increase in MLCK protein expression. SiRNA transfection was performed for 48 hr prior to LA1 or TNF-α treatments. Relative densitometry of MLCK indicated right; ^***^, *P*<0.001 vs. siNT; ^##^, *P*<0.01 vs.TNF-α; ^††^, *P*<0.01 vs. TNF-α+LA1 treatment.

MLCK is a TJ effector protein that plays a central role in the regulation of intestinal TJ barrier (102, 103). The increase in intestinal TJ permeability caused by TNF-α has been shown to be regulated in part by NF-κB p50/p65 activation of MLCK gene and increase in MLCK activity in the intestinal epithelial cells, leading to the opening and disruption of the intestinal TJ barrier (28, 30, 32, 33, 72, 102–109). The effect of LA1 on increase in MLCK gene activity, protein expression, and kinase activity in filter-grown Caco-2 monolayers caused by TNF-α was investigated. TNF-α caused an upregulation of MLCK gene activity as evidenced by the increase in the full-length MLCK promoter activity (2,091 bp promoter region, accession # DQ090939) as assessed by the luciferase reporter gene, and an upregulation in mRNA levels (**Figures 6A, B**). TNF-α also caused an upregulation in MLCK expression and kinase activity, as assessed by Caco-2 MLC phosphorylation (**Figures 6C, D**). As seen in **Figure 6D**, TNF-α produced a marked increase in phosphorylated-MLC near the per-junctional regions of filter-grown Caco-2 monolayers. The LA1 pre-treatment inhibited the upregulation of MLCK promoter activity, mRNA and protein expression, and MLC phosphorylation (**Figures 6A–D**). The inhibition of PI3K by siRNA abolished the LA1 inhibition of TNF-α increase in MLCK expression (**Figure 6E**).

### 3.7 LA1 inhibits the TNF-α–induced increase in mouse intestinal permeability *in vivo*

**Figure 7:**
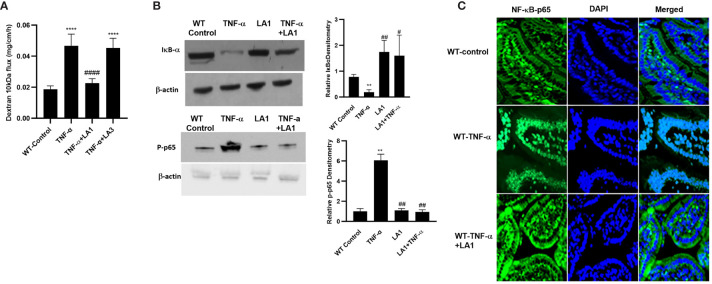
Effect of LA1 on TNF-α induced increase in mouse intestinal permeability *in-vivo*. **(A)** Oral gavage of LA1, but not LA3, (1 x 10^9^ CFU/ml) prevented the TNF-α -induced increase in dextran 10 kDa flux in mouse intestinal tissues; ****, *P*<0.0001 vs. control; ^####^, *P*<0.00001 vs. TNF-α treatment. (TNF-α (5 μg) was administered intraperitoneally). Relative densitometry of IκB-α and p-p65 indicated right; ^**^, *P*<0.01 vs. WT control; ^#^, *P*<0.05; ^##^, *P*<0.01 vs.TNF-α; **(B)** Intraperitoneal injection of TNF-α (5 μg) caused degradation of IκB-α and increase in phospho-p65, and LA1 prevented the TNF-α-induced activation of NF-κB (2-hr experimental period). **(C)** LA1 inhibited the TNF-α-induced cytoplasmic-to-nuclear translocation of NF-κB p65 (2-hr experimental period) in small intestinal tissues as determined by immunofluorescence imaging, NF-κBp65: green; Nuclei: blue; 60x (Nikon fluorescent microscopy).

**Figure 8:**
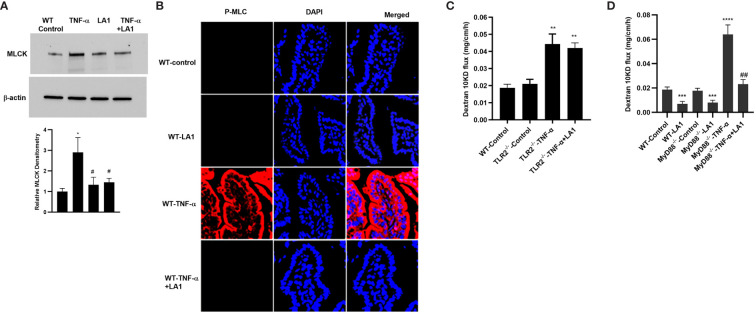
Effect of oral gavage of LA1 (1x10^9^ CFU/ml) on TNF-α-induced increase in MLCK expression and activity *in-vivo*. **(A)** LA1 prevented the TNF-α-induced increase in MLCK protein expression in mouse small intestinal tissue (24-hr experimental period). Relative densitometry of MLCK indicated below; ^*^, *P*<0.05 vs. WT control; ^#^, *P*<0.05 vs.TNF-α treatment. **(B)** LA1 inhibited the TNF-α-induced increase in MLCK activity as assessed by phospho-MLC immunostaining (24-hr experimental period); P-MLC: red; Nuclei: blue; 63x (Confocal microscopy). **(C)** LA1 did not prevent the TNF-α-induced increase in mouse intestinal permeability to dextran10 kDa in TLR-2^-/-^ mice; **, *P*<0.001 vs. wild-type (WT) control and TLR-2^-/-^ control (24-hr experimental period). **(D)** LA1 caused a decrease in mouse intestinal permeability to dextran10 kDa in wild-type (WT) and in MyD88^-/-^ mice (24-hr experimental period), and prevented the TNF-α induced increase in mouse intestinal permeability in WT and MyD88^-/-^ mice. ***, *P*<0.001 vs. WT and vs. MyD88^-/-^ control; ****, *P*<0.0001 vs. control; ^##^, *P*<0.01 vs. TNF-α treatment.

In these animal studies, we assessed the LA1 effect on TNF-α-induced increase in intestinal epithelial TJ permeability in mice. The effects of LA1 and TNF-α administration on mouse small intestinal permeability were determined *in vivo* by “recycling intestinal perfusion of an isolated small intestinal segment (6-8 cm in linear length) in live mice using Texas Red- dextran 10 kDa as the paracellular marker”, as described previously by us (71). In these studies, LA1 was administered by oral-gastric gavage 24 hr prior to the intraperitoneal (i.p.) treatment of TNF-α (5 μg for 24 hr). The TNF-α treatment caused an increase in mouse small intestinal permeability to dextran 10 kDa by the end of the 24hr treatment period (27) (**Figure 7A**). A dose-dependent effect of LA1 on mouse intestinal permeability was examined to verify the most beneficial dose of LA1 on the intestinal TJ barrier. The bacterial concentration of 1x10^9^ CFU/ml exerted the maximal effect on mouse intestinal TJ barrier (data not shown). The pre-treatment with LA1, but not LA3, inhibited the TNF-α increase in mouse intestinal permeability (**Figure 7A**). The TNF-α caused downregulation of IκB-α and an increase in NF-κB p65 phosphorylation in mouse intestinal tissue (**Figure 7B**), and LA1 pre-treatment inhibited the IκB-α degradation and NF-κB p65 phosphorylation (**Figure 7B**). The images of the mouse intestinal tissue showed that NF-κB p65 is present in the cytoplasm of the intestinal epithelial cells in the control mice (**Figure 7C**). Following TNF-α administration, NF-κB p65 translocated to the nuclei in mouse enterocytes (**Figure 7C**); the LA1 pre-treatment prevented the nuclear translocation of NF-κB p65 (**Figure 7C**). Additionally, TNF-α caused an upregulation in MLCK expression in intestinal tissues (**Figure 8A**) and an increase in phosphorylated MLC in the mouse enterocytes as shown by the immunostaining of the intestinal mucosal surface (**Figure 8B**). The requirement of TLR-2 in LA1 protective effect against the TNF-α-induced increase in mouse intestinal permeability was also determined in TLR-2^−/−^ mice. The TNF-α-induced increase in intestinal permeability was inhibited in LA1 pre-treated wild-type mice (**Figure 7A**); the LA1 inhibition of TNF-α-induced increase in mouse intestinal permeability was prevented in the TLR-2^−/−^ mice (**Figure 8C**), suggesting that TLR-2 was required for the LA1 intestinal barrier protective effect. On the other hand, LA1-induced decrease in mouse intestinal permeability was not prevented in MyD88^-/-^ mice (**Figure 8D**). And, LA1 pre-treatment prevented the TNF-α induced increase in mouse intestinal permeability in MyD88^-/-^ mice, suggesting that the LA1 effect on mouse intestinal permeability was MyD88-independent.

## 4 Discussion

Defective intestinal epithelial TJ barrier has been shown to play a role in the pathogenesis of IBD, and, in patients with IBD, therapeutic tightening of the intestinal TJ barrier correlates with rapid resolution of intestinal inflammation and prolonged clinical remission (110). Pro-inflammatory cytokine TNF-α is highly expressed in patients with IBD and plays a crucial role in the initiation, prolongation, and persistence of intestinal inflammation, and anti-TNF-α antibody therapy has been shown to be highly effective in the treatment of CD and UC (40, 42, 43). TNF-α produces a rapid and prolonged increase in intestinal TJ permeability by activating MLCK gene in intestinal epithelial cells (27, 31–33–72, 104). Intestinal microflora plays an important role in maintaining a healthy gut microenvironment and also in initiating or promoting exaggerated inflammatory responses in various disease states (87, 111, 112). Probiotic bacteria are gut bacteria that provide health benefits, including the restoration of intestinal barrier function (48, 50, 59). Most probiotic bacteria studied to date have no or modest effect on intestinal epithelial TJ barrier (113, 114). Although *Lactobacillus* species are the most widely studied probiotic bacterial species in relation to health benefits, most of the *Lactobacillus* species studied have “had a modest effect on intestinal TJ barrier” (60–63, 66–68, 70). Hsieh et al. tested over 30 probiotic bacterial species and strains, including *Lactobacillus* and *Enterococcus*, and found that the majority had no effect or caused a decrease in intestinal TJ barrier function and that *Bifidobacterium* strains produced a modest increase (115). Lepine et al. showed that *L. acidophilus* strain (W37) caused a modest enhancement in Caco-2 TJ barrier (64), and Guo et al. found that another strain of *Lactobacillus*, *L. acidophilus* strain (ATCC No. 53103) produced a 25% increase in Caco-2 TER (62). Madsen et al. (116) and Mennigen et al. (113) showed that probiotic mixture VSL#3 (containing nine probiotic bacterial species, including *L. acidophilus*) also caused a modest enhancement in mice and human intestinal epithelial barrier function. Similarly, in our previous studies, we also found that many of the over 20 probiotic species tested, including *Lactobacillus plantarum*, *L. brevis*, *L. helveticus*, *L. casei*, and *Bifidobacterium longum* had no effect on Caco-2 TJ barrier and several probiotic species, including *L. johnsonii*, *L. rhamnosus* and *B. breve* had a modest effect (10-25% increase) on Caco-2 TER. However, there was one particular strain of LA that caused an optimal enhancement in intestinal epithelial TJ barrier function, while other LA strains tested “had a modest or no effect on the intestinal TJ barrier” (71). We found two strains of LA, referred to as LA1 and LA3, that had distinct effects on intestinal epithelial TJ barrier in Caco-2 monolayers and in mice small intestine (71). LA1 caused a marked enhancement in intestinal epithelial TJ barrier function, while LA3 had no effect on the intestinal TJ barrier (71). LA1 concentration of 1x10^8^ CFU/ml and 1x10^9^ CFU/ml exerted the maximal enhancement of intestinal TJ barrier in Caco-2 cells and in mouse intestinal tissues, respectively. Additionally, LA1, but not LA3, inhibited the development of DSS-induced colitis and prevented intestinal inflammation by preserving the intestinal barrier (71). The anti-inflammatory and antioxidant mechanisms involved in different strains of LA related to the enhancement of the intestinal barrier have been examined (57, 117–120). For example, a study by Chorawala et al. showed that LA preserve the intestinal TJ barrier by exerting anti-inflammatory response against LPS-induced colitis (117). The study also showed the antioxidant effect of LA and the enhancement of the barrier on colonic cells in mouse model of colitis. The colonic C-reactive protein, nitric oxide and pro-inflammatory cytokines were all reduced after exposure to LA and other probiotic strains (117). De Matuoaka et al. also showed the potential role of LA strains as protective agents against arsenic toxicity and arsenic-induced intestinal barrier disruption in Caco-2 cells (118). The main aim of the present study was to investigate a novel intracellular mechanism that mediate the LA1 inhibition of TNF-α-induced increase in intestinal epithelial TJ permeability. The findings in the present study expand on our previous studies showing that “LA1 causes a unique, strain-specific enhancement of intestinal barrier function” (71) and provide new insight into the signaling pathways and the mechanisms by which LA1 inhibits the TNF-α-induced increase in intestinal TJ permeability. Our results suggest for the first time that LA1 inhibits the TNF-α increase in intestinal TJ permeability via a novel intracellular process in which LA1 suppresses the NF-κB p50/p65 activation in a TLR-2-dependent and MyD88-independent mechanisms in the intestinal epithelial cells.

NF-κB p50/p65 is a ubiquitous pro-inflammatory nuclear transcription factor that mediates the activation of immune response and plays a central role in promoting intestinal inflammation in IBD and other inflammatory conditions of the gut (7, 83, 84). The NF-κB activation is separated into canonical and non-canonical pathways: the canonical pathway leads to the activation of NF-κB p50/p65 (RelA) heterodimer, and the non-canonical pathway leads to the activation of NF-κB p52/RelB heterodimer (28, 85, 121). The canonical pathway activation of NF-kB p50/p65 is known to be regulated by the IKK complex consisting of the catalytic subunits IKK-α and IKKβ and the regulatory subunit IKK-γ (122–124). The IKK catalytic subunits enzymatically catalyze the phosphorylation, ubiquitination, and degradation of IκB-α protein and the concomitant NF-κB p50/p65 activation (124, 125). The non-canonical pathway is classically regulated by the up-stream NIK activation of IKK-α homodimer and the subsequent phosphorylation and proteolytic processing of p100 and production of p52 and RelB (122, 124, 126). The TNF-α induced increase in intestinal TJ permeability has been shown to be mediated by the activation of the canonical pathway NF-κB p50/p65 in intestinal epithelial cells via a newly described intracellular mechanism, in which the NIK (typically associated with the non-canonical pathway p52/RelB activation) sequentially activates IKK-α and NF-κB p50/p65 (28). In the studies herein, we show that LA1 by itself does not affect the basal levels of NF-kB p50/65 activation in intestinal epithelial cells but inhibits the TNF-α activation of NF-κB p50/p65 and the subsequent increase in intestinal TJ permeability in a TLR-2 dependent mechanism. Toll-like receptors (TLRs) are pattern recognition receptors (PRRs) that play a crucial role in innate immunity by recognizing various PAMPs in response to microbe invasion (127). The current scientific paradigm is that, in response to a microbe invasion, TLRs, including TLR-2, TLR-4, and TLR-5, recruit and employ adaptor protein MyD88 to activate NF-κB p50/p65 and stimulate immune response (76, 128–130). In our studies herein, LA1 also produced a rapid activation of NF-κB p50/p65 in immune cells in a TLR-2 and MyD88-dependent manner (**Figure 3**). Although the prior studies have uniformly found TLR signal transduction pathways, including TLR-2, TLR-4, and TLR-5, to activate NF-κB p50/p65 in immune cells and other cell types, our results in intestinal epithelial cells demonstrated that LA1 inhibits the NF-κB p50/p65 activation in a TLR-2 dependent process and that TLR-2 knock-down abolished the LA1 suppression of NF-kB activity (**Figure 4**). This shows a unique mechanism of LA effect on intestinal epithelial cells distinct from the immune response. It is also interesting to note that the LA1/TLR-2 pathway suppression of intestinal epithelial NF-κB p50/p65 was independent of MyD88 (**Figure 4E**), a known master regulator of NF-κB activation (128–132). In combination, our results showed a novel finding that TLR-2 complex has a differential regulatory role in NF-κB activation depending on the cell-type, and that LA1/TLR-2 signal transduction pathway activates NF-κB p50/p65 in immune cells, whereas it inhibits the NF-κB activation in intestinal epithelial cells. Since intestinal epithelial cells are at the interface of the outside and the internal milieu of the body and serve as a physical and functional barrier that selectively regulates the transport of needed nutrients yet simultaneously restricts the trans-epithelial uptake of noxious substances and gut bacteria in the intestinal lumen, it would be phylogenetically disadvantageous and detrimental in the evolution of organisms to have luminally accessible apical membrane receptors that trigger inflammatory response or lead to the disruption of the intestinal barrier function in the presence of gut bacteria. On the other hand, having apical membrane receptors such as TLR-2 accessible to commensal or probiotic bacteria and which can be regulated to enhance the intestinal TJ barrier or suppress inflammatory response (up-regulate immune tolerance) would be evolutionally advantageous (133, 134).

As for the crosstalk between TLR-2 and TNF-α signal transduction pathways and the intermediatory protein kinase that modulates the NF-κB activation process, our data demonstrated that LA1 caused PI3K activation in intestinal epithelial cells in a TLR-2-dependent process, and that PI3K plays an important regulatory role in NF-κB p50/p65 inhibition by interfering with IKK-α phosphorylation (**Figure 5E**). The increase in intestinal TJ permeability caused by TNF-α has been found to be mediated by NF-κB p50/p65 binding and activation of enterocyte MLCK promoter and MLCK-induced opening of the TJ barrier (28–31, 34, 104). Our results herein also show that LA1 inhibits the TNF-α induced increase in intestinal permeability by PI3K-dependent inhibition of MLCK gene activation and MLCK activity in Caco-2 cells and mouse enterocytes (**Figures 6**, **8**). These results suggest that TLR-2 complex and PI3K are potential therapeutic targets to inhibit enterocyte NF-κB p50/p65 activation and MLCK activity and to preserve the intestinal TJ barrier in various intestinal inflammatory disorders.

In conclusion, our results show that LA1 inhibits the TNF-α-induced increase in intestinal epithelial TJ permeability via a TLR-2-dependent inhibition of NF-κB p50/p65 activation. LA1 produces a TLR-2- and MyD88-dependent activation of NF-κB p50/p65 in immune cells and TLR-2-dependent, but MyD88-independent, suppression of NF-κB activation in intestinal epithelial cells, demonstrating that LA1 activation of TLR-2 signal-transduction pathway has a cell-type specific and differential regulatory effect on NF-κB p50/p65 activation. The LA1/TLR-2 and TNF-α/TNF-α-receptor signaling pathway crosstalk was mediated in part by PI3K inhibition at the level of IKK-α and the subsequent down-stream inhibition of enterocyte NF-κB p50/p65 and MLCK gene activity. In composite, these data provide important and novel insight into the role TLR-2 and PI3K play in cell-type specific inhibition of TNF-α-induced activation of NF-κB p50/p65 and increase in intestinal TJ permeability.

## Data availability statement

The raw data supporting the conclusions of this article will be made available by the authors, without undue reservation.

## Ethics statement

Ethical approval was not required for the studies on humans in accordance with the local legislation and institutional requirements because only commercially available established cell lines were used. The animal study was approved by Penn State University Animal Care and Use Committee. The study was conducted in accordance with the local legislation and institutional requirements.

## Author contributions

MH: Formal analysis, Investigation, Methodology, Validation, Visualization, Writing – review & editing. LK: Formal analysis, Investigation, Methodology, Validation, Visualization, Writing – review & editing. RA: Formal analysis, Methodology, Writing – review & editing. JE: Methodology, Writing – review & editing, Validation. EK: Methodology, Formal analysis, Validation, Writing – review & editing. MR: Methodology, Writing – review & editing. RA-S: Conceptualization, Project administration, Supervision, Validation, Writing – review & editing, Methodology, Formal analysis, Investigation, Visualization, Writing – original draft. TM: Conceptualization, Funding acquisition, Project administration, Resources, Supervision, Validation, Writing – review & editing.
